# Smooth Muscle Transcriptome Browser: offering genome-wide references and expression profiles of transcripts expressed in intestinal SMC, ICC, and PDGFRα^**+**^ cells

**DOI:** 10.1038/s41598-018-36607-6

**Published:** 2019-01-23

**Authors:** Adrienne Breland, Se Eun Ha, Brian G. Jorgensen, Byungchang Jin, Treg A. Gardner, Kenton M. Sanders, Seungil Ro

**Affiliations:** Department of Physiology and Cell Biology, University of Nevada School of Medicine, Reno, Nevada USA

## Abstract

Transcriptome data on the quantitative numbers of transcriptional variants expressed in primary cells offer essential clues into specific cellular functions and biological processes. We have previously collected transcriptomes from primary smooth muscle cells (SMC), interstitial cells of Cajal (ICC), and PDGFRα^+^ cells (fibroblast-like cells) isolated from murine jejunal and colonic smooth muscle and/or mucosal tissues as well as transcriptomes from the associated tissues (jejunal smooth muscle, colonic smooth muscle, and colonic mucosa). In this study, we have built the Smooth Muscle Transcriptome Browser (SMTB), https://med.unr.edu/physio/transcriptome, a web-based, graphical user interface that offers genetic references and expression profiles of all transcripts expressed at both the cellular (SMC, ICC, and PDGFRα^+^ cells) and tissue level (smooth muscle and mucosal tissue). This browser brings new insights into the cellular and biological functions of the cell types in gastrointestinal smooth muscle biology.

## Introduction

The proper regulation of gut activity is vital to homeostasis and survival. The digestive process of absorbing nutrients and releasing them into the bloodstream is achieved through a series of synchronized involuntary movements (motility) of gastrointestinal (GI) smooth muscle, which mixes food and propels the digested content through the GI tract^1^. Smooth muscle tissue is comprised of a diverse range of unique cellular subpopulations that require isolation for individual study to aid in the elucidation of each subpopulations contribution to the functioning of the overall tissue. Motility in the GI tract is controlled by several types of cells including smooth muscle cells (SMC), interstitial cells of Cajal (ICC), PDGFRα^+^ cells (fibroblast-like cells), as well as the enteric nervous system (ENS)^1^. ICC generate spontaneous electrical slow waves^2^, the ENS generates complex rhythmic motor behavior^3^, and PDGFRα^+^ cells mediate enteric inhibitory responses^4,5^, all of which control SMC, the final effectors for muscle contraction and muscle relaxation^1^. The three cell types, SMC, ICC, and PDGFRα+ cells (SIP cells), are electrically coupled via gap junctions and create an electrical syncytium, which collectively regulate GI motility^1^. Developmental abnormalities and pathophysiological damage to these cells are directly linked to GI neuromuscular diseases such as Hirschsprung’s disease^6^, diabetic gastroenteropathy^7^, gastrointestinal stromal tumor^8^, intestinal fibrosis^9^, and chronic intestinal pseudo-obstruction^10^. All of these motility diseases are thought to be developed from the remodeling of the smooth muscle in the GI tract, leading to abnormal growth (hypertrophy or tumor), myopathy, or death of the cells.

Genome-scale expression profiles of specific cell types provide indispensable information regarding cellular identity and function. To access the genetic information of SMC, ICC, and PDGFRα^+^ cells within the small intestine and colon, we launched a Smooth Muscle Transcriptome Sequencing Project. For this project, we isolated primary jejunal and colonic SMC, ICC, and PDGFRα^+^ cells (mucosa and muscularis) from cell-specific GFP reporter mouse lines, and obtained a transcriptomic profile of each cell type and associated tissue^11–14^. In analyzing each cell type’s transcriptome, we identified new markers and signature genes for each cell type that are linked to cellular functions^11–14^.

To help to explore this complex dataset, we built the SMTB. This graphical, web-based, browser displays the comprehensive expression profile and genomic map of each cell type and associated tissue within the colon and jejunum. The browser is available online, hosted by the University of Nevada, Reno at https://med.unr.edu/physio/transcriptome. This resource provides genome-wide genetic references and expression levels, enabling insight into genetic structure, expression profile, and isoforms of each gene expressed in key GI cell and tissue populations.

## Results

The SMTB offers genome-wide genetic references and unique graphical images that can reveal new insights into the genetic structures, expression profiles, and isoforms of each gene expressed in key GI cell populations (SMC, ICC, and PDGFRα^+^ cells) and GI tissues (jejunum SM, colonic SM and mucosa) for functional studies.

## Applications

- Expression levels of various genes within GI tissues and GI cell types.
- Expression levels, and numbers, of transcriptional gene variants in GI tissues and GI cell types.
- Observing genomic structure (promoter, exons and introns) of transcriptional variants.
- Primer design for RT-PCR or qPCR (designing primers to span exon to exon junctions in order to minimize genomic DNA amplification and to detect specific transcriptional variants).
- Viewing splicing donor and acceptor sequence sites of transcriptional variants.
- Obtaining cDNA sequences for transcriptional variants.
- Finding open reading frames within transcriptional variants.

## User's guide

The SMTB is accessible at https://med.unr.edu/physio/transcriptome/smooth-muscle-transcriptome-browser.

1. Once arrived at the home page, click “Access the Smooth Muscle Transcriptome Browser” to take you to the browser.
2. Go to “Select Track” as shown in Fig. 1a. There are two references of the mouse genome, mm9 (NCBI37, July 2007) and mm10 (GRCm38, Dec. 2011). Select one reference from the “Reference” section. As an example, mm9 was selected in Fig. 1a. Under the transcripts section, there are seven cell types (SMC Jejunum, ICC Jejunum, PDGFRαC Jejunum, SMC Colon, ICC Colon, PDGFRαC Colon, and PDGFRαC Mu Colon), three tissue types (SM Jejunum, SM Colon, and Mu Colon), and combined transcripts (GI All). Select cell(s)/tissue(s) as interested. For example, SMC Colon and SMC Jejunum were selected (Fig. 1a). Once all selections have been made, click “Back to Browser”.

**Figure 1:**
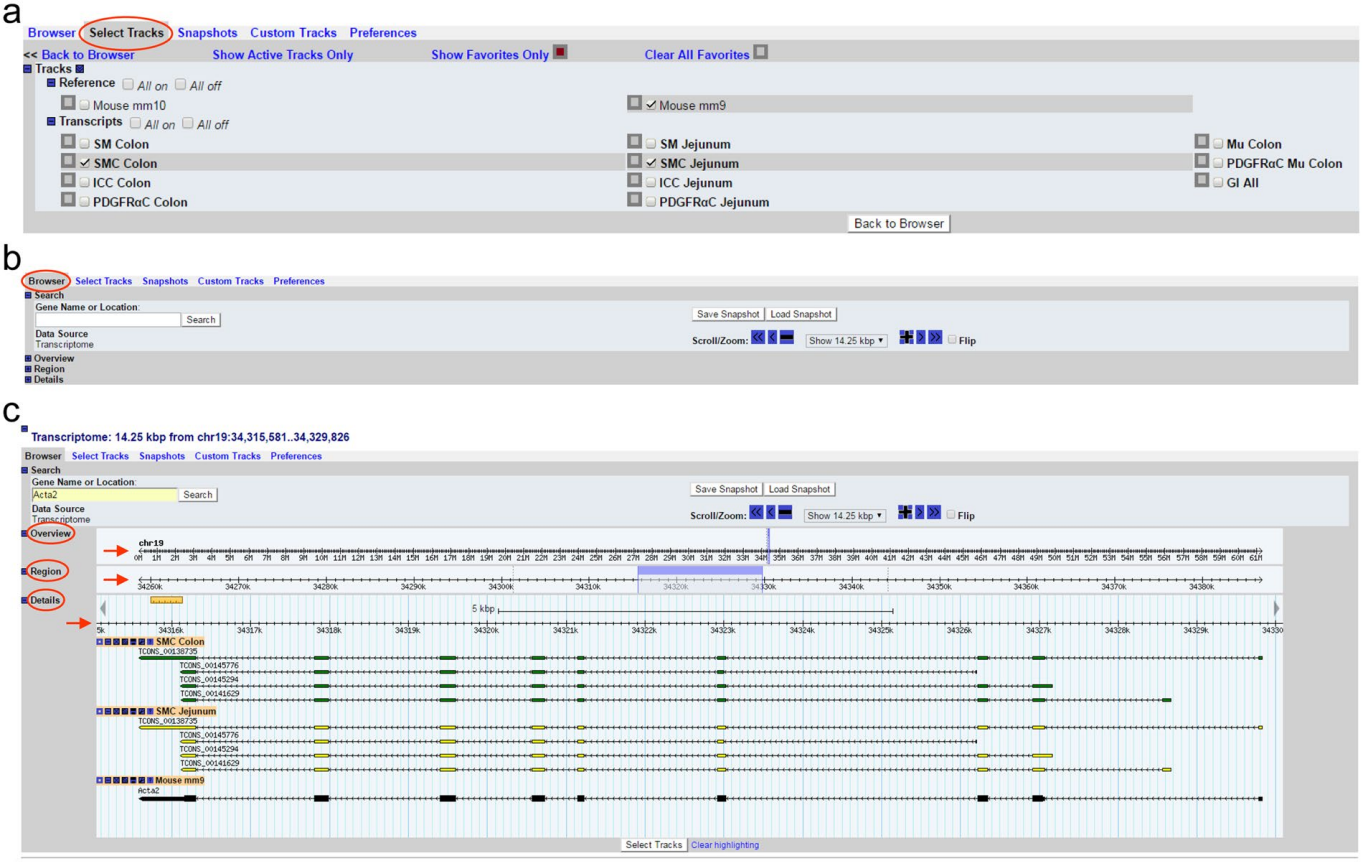
Smooth Muscle Transcriptome Browser built with Gbrowse 2.0. (**a**) “Select Tracks” tab showing the two selectable mouse reference genomes, mm9 and mm10, as well as the various selectable transcripts from each cell type and tissue. (**b**) The home screen of the “Browser” tab. (**c**) The search result of Acta2 in the “Browser” tab. Shown are the “Overview,” “Region,” and “Details” section showing the chromosomal location, a highlighted region marker (light blue), and a graphical representation of *Acta2* transcriptional variants expressed in colonic (green) and jejunal (yellow) SMC. A map of the reference gene is also shown under the transcripts (black).
3. The browser itself contains sections include “Search,” “Overview,” “Region,” and “Details” (Fig. 1b). Type a gene name in “Gene Name or Location” under the Search section (overwrite the chromosome location) and click “Search.” As an example, Acta2 (aka alpha smooth muscle actin) was typed in and searched for (Fig. 1c). The browser retrieves and displays the genomic map of Acta2 and the transcriptional variants expressed in both colonic and jejunal SMC. In the “Overview” and “Region, it displays the location of Acta2 on chromosome 19 with the zoomed in location highlighted in a light blue bar in the “Region” section. This bar can be clicked and dragged to shift the displayed chromosomal location. In the “Details” section, the transcriptional maps of four isoform variants expressed in colonic and jejunal SMC are displayed. Exons in the variants are marked by green (colonic SMC) or yellow (jejunal SMC) boxes. For Acta2, the directionality of the arrows on the lines of each variant indicates that the cDNAs match to the antisense strand. Among the four Acta2 transcriptional variants, TCONS_00138735 is the longest in both colonic and jejunal SMC which is the same as the reference transcript of *Acta2* shown in black (Fig. 1c). Wider boxes on the line of the reference gene mark a coding cDNA region while narrow boxes show 5′ and 3′ non-coding regions.
4. The genomic view can be zoomed in and out on any chromosomal location (Fig. 2). Fixed ranges for zooming are from 100 bp to 1 Mbp (Fig. 2a). In addition, zoom levels can be changed in 10% increments using “+” or “−” buttons in the “Scroll/Zoom” section just above the browser. Furthermore, the view can be freely moved to left or right by clicking any of the “≪”, “<”, “◄” (to left), “≫”, “>”, “►” (to right) buttons, or by holding and dragging the light blue bar in either direction (Fig. 2b). Chromosomal location and distance can be displayed and measured by clicking the ruler and dragging to the area of interest (Fig. 2b). The reference genes displayed are linked to Gene Search Results in NCBI showing a list of a related gene associated with a full gene report when clicked (Fig. 2b).

**Figure 2:**
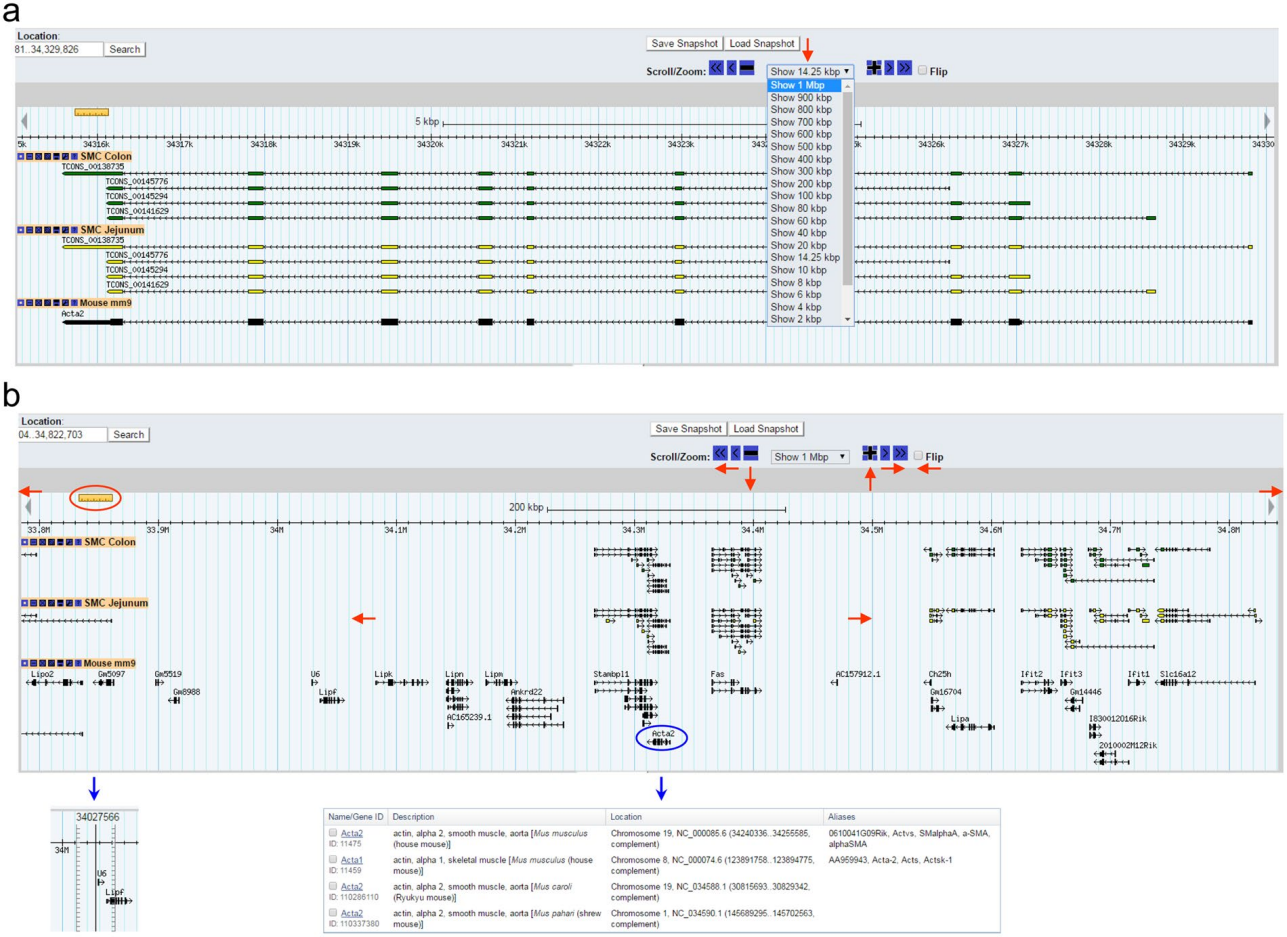
A map view of *Acta2* transcripts in the genome. (**a**) Zooming capabilities range from 1 Mbp to 100 bp at the *Acta2* locus (red arrow). (**b**) A genomic view of 1 Mbp around the *Acta2* gene locus. This map view can be freely moved left (←), or right (→) in the genome by the “Scroll/Zoom” menu options of “≪”, “<”, “◄”, “≫”, “>”, or “►”. Zoom is controlled by “−” or “+” or out (↓) and in (↑). “Flip” allows the user to switch between sense strand to anti-strand view or vice versa. Reference genes on the view are linked to the NCBI (*Acta2*, encircled in blue, is linked to reference genes at the NCBI). The ruler on left can be moved and denotes an exact chromosomal location of the cursor.
5. The genomic map image of Acta2 can be saved in “Snapshots” by clicking the “Save Snapshot” button just above the “Scroll/Zoom” section (Fig. 3a). The saved image can be reloaded, removed, or stored in the “Snapshots” tab above the browser by clicking it (Fig. 3b). The stored image can be also downloaded (Fig. 3c).

**Figure 3:**
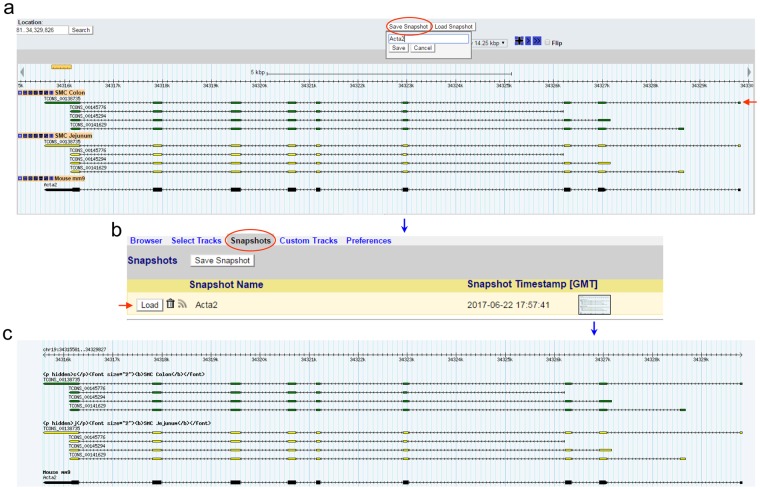
Saving and reloading a map of *Acta2*. (**a**) The map of *Acta2* to be saved as Acta2 by clicking “Save Snapshot”. (**b**) An image of the map view saved under the “Snapshots” tab. This view can be reloaded by “Load” or removed by clicking the trash can. (**c**) A saved image of *Acta2*. The reloaded view (**a**) shows four transcriptional variants found in “SMC Colon” and in “SMC Jejunum”. The longest variant is TCONS_00138735 and is indicated by a red arrow in (**a**).
6. Each transcriptional variant on the map is linked to detailed data. The longest transcriptional variant of Acta2, TCONS_00138735, will be used as an example for analysis (Fig. 3a). By clicking on the structural display of TCONS_00138735 under the “Details” section, you will be linked to a page containing a summary of exonic numbers and locations, expression profiles, and cDNA sequence (Fig. 4). Exonic number, length, and associated chromosomal locations are summarized in Fig. 4a. Each exon contains a hyperlink to a map view of that exon. Expression levels (FPKM) of TCONS_00138735 in jejunal cells (JSMC, JICC, and JPαC), jejunal muscle tissue (JSM), colonic cells (CSMC, CICC, and CPαC), colonic muscle tissue (CSM), colonic mucosal PDGFRα+ cells (CMuPαC), and colonic mucosal tissue (CMu) are shown in as various histograms (Fig. 4b). This variant is dominantly expressed in JSMC and CSMC. It is also noticeably expressed in JPαC and CMuPαC. Total expression levels of all four transcriptional variants of *Acta2* are shown in Fig. 4c. Figure 4d shows the DNA sequence of TCONS_00138735 containing 9 exons and 8 introns. The cDNA sequence can be downloaded as a.doc file by clicking the “Download cDNA Sequence” hyperlink above the displayed sequence. In addition, the cDNA can be further analyzed to search open reading frames (ORF).

**Figure 4:**
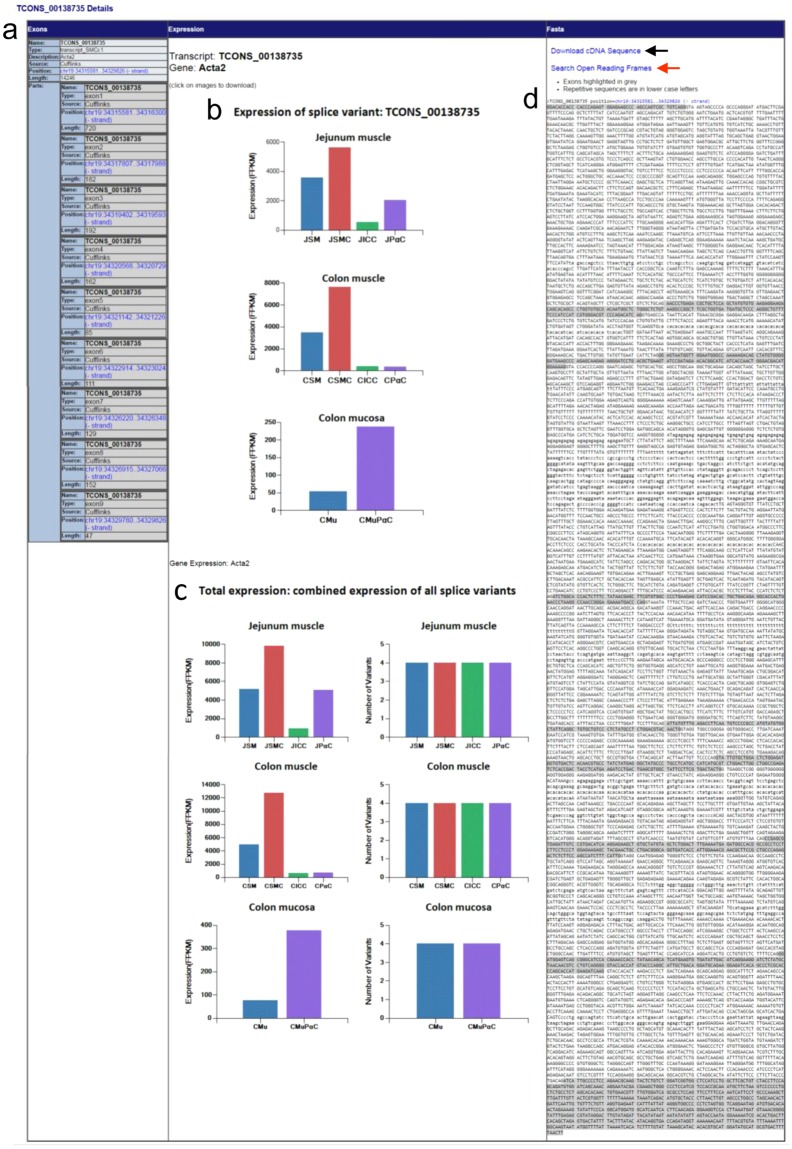
The expression profile and exon/intron structure of *Acta2* TCONS_00138735. (**a**) Summary of the exonic map of TCONS_00138735. The number of exons and respective chromosomal locations are shown. Each exon position (hyperlinked in blue) is linked to the map view. (**b**) Expression level (FPKM) of TCONS_00138735 from *Acta2* in jejunal and colonic cells as well as tissues. The image can be downloaded into as a.gif file and saved. (**c**) Total expression level (FPKM) and the number of all transcriptional variants of *Acta2* found in jejunal and colonic cells and tissues. The image can be downloaded into a.gif file and saved. JSM, jejunal smooth muscle; JSMC, jejunal SMC; JICC, jejunal ICC; JPαC, jejunal PDGFRα^+^ cells; CSM, colonic smooth muscle; CSMC, colonic SMC; CICC, colonic ICC; CPαC, colonic PDGFRα^+^ cells; CMu, colonic mucosa; CMuPαC, colonic mucosal PDGFRα^+^ cells. (**d**) DNA sequence of TCONS_00138735. Exons are highlighted on the sequence in grey. Repeated sequences (aka repetitive elements) are written in lowercase. TCONS_00138735 cDNA sequence can be downloaded and saved as a. Doc file as indicated by a black arrow. It is also linked to the ORF Finder at NCBI by clicking “Search Open Reading Frames” (a red arrow).
7. Instructions for searching ORF is shown in Fig. 5. Acta2 TCONS_00138735 cDNA is 1,780 bp in length. The cDNA sequence was copied, pasted, and submitted in the Enter Query Sequence in the NCBI ORF Finder^15^ (Fig. 6a). The search produced 12 possible ORFs (ORF1-12, Fig. 6b). ORF1 is the longest one with 1,134 bp (377 amino acids) stating at nucleotide (nt) 71 to nt 1,204. The amino acid sequence can be further analyzed by protein BLAST (SMARTBLAST or BLAST) searching for homologous proteins.

**Figure 5:**
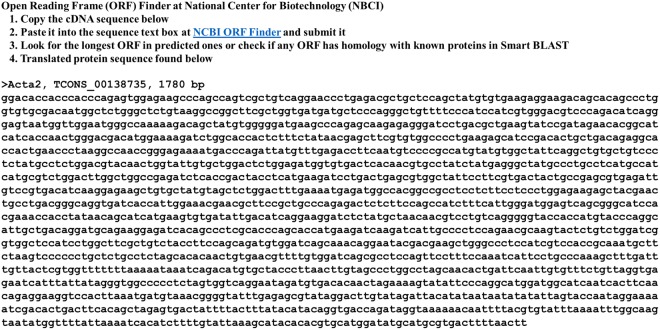
The cDNA sequence of TCONS_00138735 and instructions on using the Open Reading Frame (ORF) Finder at NBCI.

**Figure 6:**
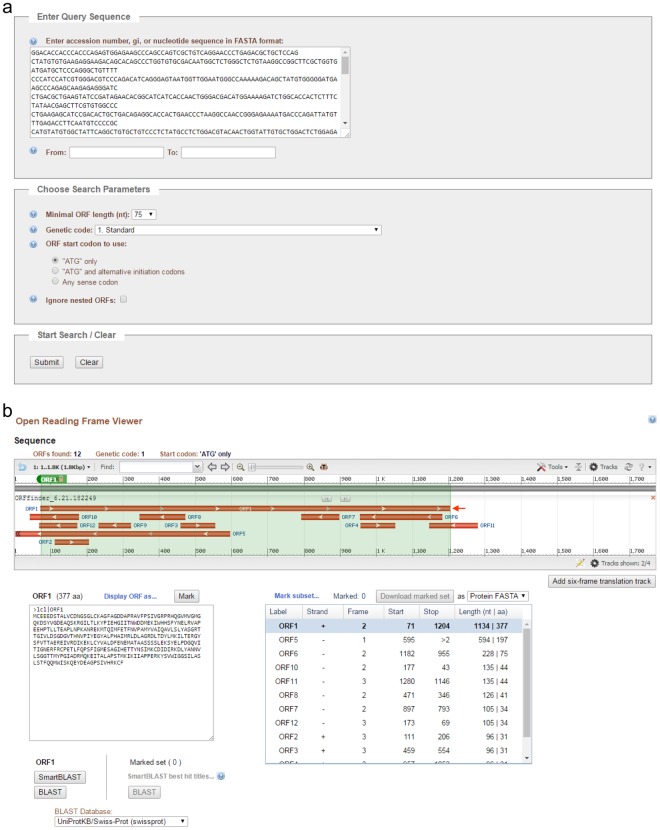
Submission of TCONS_00138735 cDNA to ORF Finder and selection of a putative ORF. (**a**) A snapshot of TCONS_00138735 cDNA pasted in ORF Finder. (**b**) A snapshot of the ORF Viewer showing TCONS_00138735 ORFs. The longest ORF, ORF1, is indicated by an arrow. Translated ORF1 amino acid (aa) sequence and summary of all ORFs are shown.

## Discussion

The Smooth Muscle Transcriptome Browser (SMTB) built in this study provides both the number and expression level of each transcriptional variant for all mapped genes expressed in SIP cells (SMC, ICC, and PDGFRα^+^ cells) within the mucosa and muscularis layers of the jejunum and colon. The cellular transcriptomes and expression profiles are provided along with the expression profiles of each cell’s respective associated tissue including the jejunal muscularis, colonic muscularis, and mucosa. This browser allows researchers to analyze each transcriptional variant in terms of gene structure (promoter region, exons, and introns), expression levels within each cell type and tissue, and open reading frames (coding protein) within each variant.

We previously built UCSC Smooth Muscle Genome Browser (SMGB)^11^ that can interact with our SMC transcriptome, SRF (SMC-specific transcription factor) binding CArG boxes, and the ENCODE data contained within the UCSC Genome Browser^16^. Later, we added the transcriptomes of ICC, and PDGFRα^+^ cells from both the muscularis and mucosa to our browser^12–14^. This interactive database can analyze genetic/epigenetic structures and the regulation of individual genes expressed in SIP cells by integrating the abundant bioinformatics data in the UCSC Genome Database^17^. Now, as this paper has reported, we have built the SMTB that is capable of searching the expression profile of individual transcriptional variants of all mapped genes expressed in SIP cells, something that was not available with any of our previously reported SIP cell transcriptome studies. The two browsers serve as cooperative and powerful tools for use in functional studies of SIP cells.

As an example, the *Acta2* gene was chosen for analysis using the SMGB and SMTB (Fig. S1). Both browsers can carry the mouse mm9 genome showing the same region chr19: 34,315,581–34,329,826 (14,246 kbp), within which four transcriptional variants, V1–4, of the gene are expressed in JSMC and CSMC. Within the SMGB, SRF binding CArG boxes, ENCODE data (SRF, H3K27ac, Pol2, JunD, and c-Jun binding sites, as well as DNase I hypersensitivity) were selected (Fig. S1a). Both V1 and V2 start in the proximal promoter regions that contain two SRF binding sites, within which match two conserved CArG boxes for each variant (CCATATAGGG and CCAAACAAGG for V1, CCATATTTAG, CCTAATTAGG for V2). The region around the SRF binding sites coincide with H3K27ac and Pol2 sites (Fig. S1a), suggesting that the gene is active, and V1 and V2 are transcribed by Pol2 while being regulated by SRF (due to the presence of SRF binding sites and CArG boxes) in intestinal SMC. In addition, a binding site for the transcription factor subunits JunD and c-Jun can be found within intron 7, which also matches a DNase I hypersensitive region (Fig. S1a), suggesting the transcription of V1-4 could be regulated by the Jun family in JSMC and CSMC. The corresponding region (240 bp, chr19: 34,318,625–34,318,864) of the Jun binging site was located on SMTB (Fig. S1b), the sequence of 240 bp was analyzed for the presence of Jun binding sites in the transcriptional regulatory element search database, “PROMO”^18^. This search identified two binding sequences, TATGTCA and GAAGTCA, for JunD and c-Jun (Fig. S1b). Next, expression levels of the four variants of *Acta2* were found on SMTB. V1 and V2, likely regulated by SRF, show selective high expression in JSMC and CSMC, compared to colonic and jejunal ICC and PDGFRα^+^ cells while V3 and V4 are dominantly expressed in CSMC and Jejunal PDGFRα^+^ cells (Fig. 2a). V1, V2, and V4 are also expressed in mucosa PDGFRα^+^ cells at relatively low levels. Each cDNA sequence for V1–4 was downloaded and an open reading frame (the longest one) was obtained from the NCBI ORF Finder^15^. Alignment of their amino acid sequences is shown in Fig. S2b, V1, V2, and V3 encode the full length of 377 amino acids which contains six post-translationally modified residues at N-terminus as shown in UniProt^19^. V1 encodes a truncated protein of 257 amino acids at the N-terminus that misses the six residues. As all of this integrated information shows, both the SMGB and SMTB are interactive browsers that provide genetic and epigenetic references that can be incorporated together for further functional studies in intestinal SIP cells and tissues.

While our transcriptome browser SMTB does share some similar features with other previously published genome/transcriptome browsers^20,21^, SMTB contains a myriad of analyses that these resources do not contain the entirety that SMTB encompasses. SMTB allows for graphical representation of FPKM values and number of transcripts at whole tissue (smooth muscle and mucosa) and individual SIP cell type (SMC, ICC, and PDGFRα^+^ cells) levels as well as being able to download cDNA sequences for both the mm9 and mm10 murine genome. Other browsers will allow for visualizations of FPKM data, such as the well-designed and valuable Brain RNA-seq project^21^, but it only uses a single genome reference (mm9), does not allow for cDNA sequence download or visualizations of alternatively spliced transcripts and has no overall FPKM for the entire tissue, only individual cell types. In contrast, a tissue reference genome, such as the RETINAL genome^20^, only contains transcriptomic information from the total retinal tissue and not individual cell types and does not have graphical representations or cDNA sequence downloads and only uses a single genome reference. RNA-seq data from both the small and large intestine (colon) are also available through ENCODE (RNA-seq from LICR and UW, respectively) at the UCSC Genome Browser^17^. These RNA-seq data were obtained from whole small or large intestine tissue consisting of smooth muscle and mucosa which are functionally distinct and contain a myriad of different cell types. In contrast, we separated jejunal and colonic smooth muscle from their respective mucosa and mRNA from each was independently sequenced and deposited in the SMTB. Furthermore, while our transcriptome browser is definitively tissue specific to GI SIP types, it contains a plethora of data and user-friendly capabilities that have not been contained within one specific browser to date.

For all our previously reported transcriptomes, we obtained and analyzed the RNA-seq data from SIP cells contained within the murine jejunum and colon (Table 1) that unveiled a plethora of genetic information at the cellular levels. This report is the first to compile and compare these transcriptomes. SIP cells express anywhere from 15,192–17,172 known genes (Table 2), which account for 66–75% of all known mouse genes. In addition, these genes are transcribed into multiple transcriptional variants through alternative start sites and splicing. For example, the L-type Ca^2+^ channel *Cacna1c* is expressed into 22 different variants in jejunal and colonic SMC^22^. The average number of transcriptional variants per gene is 3 (Table 2). Most variants appear to be cell-specific. Further characterization of each variant is required to explore localization of variants in subpopulations of SIP cells (e.g. longitudinal SMC, circular SMC, intermuscular ICC/PDGFRα^+^ cells, and intramuscular ICC/ PDGFRα^+^ cells), as well as to further understand the functional role of variants (if they are translated into protein or if they are pseudo-substrates for microRNAs).

**Table 1:** List of intestinal tissues and cells used for transcriptome studies.

Name	Description	Mouse strain	Background	Age	Molecule
SM_Jejunum	Jejunum SM	B6.129S7-Kittm1Rosay/J	C57BL/6	4 wks	mRNA
SMC_Jejunum	SMC in jejunum SM	B6.Cg-Tg(Myh11-cre,-EGFP)2Mik/J	C57BL/6	4 wks	mRNA
ICC_Jejunum	ICC in jejunum SM	B6.129S7-Kittm1Rosay/J	C57BL/6	4 wks	mRNA
PaC_Jejunum	PDGFRα^+^ cells in jejunum SM	B6.129S4-Pdgfratm11(EGFP)Sor/J	C57BL/6	4 wks	mRNA
SM_Colon	Colon SM	B6.129S7-Kittm1Rosay/J	C57BL/6	4 wks	mRNA
SMC_Colon	SMC in colon SM	B6.Cg-Tg(Myh11-cre,-EGFP)2Mik/J	C57BL/6	4 wks	mRNA
ICC_Colon	ICC in colon SM	B6.129S7-Kittm1Rosay/J	C57BL/6	4 wks	mRNA
PaC_Colon	PDGFRα^+^ cells in colon SM	B6.129S4-Pdgfratm11(EGFP)Sor/J	C57BL/6	4 wks	mRNA
Mu_Colon	Colon mucosa	B6.129S4-Pdgfratm11(EGFP)Sor/J	C57BL/6	4 wks	mRNA
PaC_Mu_Colon	PDGFRα^+^ cells in colon mucosa	B6.129S4-Pdgfratm11(EGFP)Sor/J	C57BL/6	4 wks	mRNA

**Table 2:** Summary of transcriptome data obtained from intestinal tissues and cells.

Name	Total read	Mapped read	Known gene	Total isoform	Average isoform
SM_Jejunum	238,246,980	184,746,380	15,837	53,337	3.4
SMC_Jejunum	150,702,388	139,478,151	15,427	46,299	3.0
ICC_Jejunum	206,711,208	177,168,420	17,172	55,259	3.2
PaC_Jejunum	149,419,352	135,517,106	16,270	48,974	3.0
SM_Colon	212,744,016	164,801,684	16,359	55,345	3.4
SMC_Colon	151,445,926	139,645,488	15,192	47,275	3.1
ICC_Colon	193,155,892	168,384,646	16,820	54,488	3.2
PaC_Colon	136,563,090	123,172,778	15,714	48,894	3.1
Mu_Colon	168,835,236	153,208,218	15,933	52,113	3.3
PaC_Mu_Colon	154,151,508	141,592,530	15,777	51,282	3.3

*GI_All: all transcriptional variants expressed in intestinal tissues and cells above.

The transcriptomes of SIP cells are very similar, sharing not only the genes that are expressed in all three types (around 93%), but also showing comparable expression levels for these gene transcripts. These similar transcriptomic profiles lend credence to the notion that these three cell types might have a shared developmental lineage arising from mesenchymal cells^23^. In contrast to their largely shared expression profiles, SIP cells also have ~1,500 genes that are unique to each cell within the syncytium. These cell type-specific genes may contribute to the individual phenotypic identity, and unique functioning, of each cell type in the SIP syncytium.

Through previous gene ontology (GO) term analysis of the predominately expressed genes within SIP cells, we found the main functional roles of each cell type. SMC genes showed unique GO terms related to muscle contraction including genes related to the cytoskeleton, actin binding, calcium ion binding, myosin complex, and smooth muscle contractile fibers^11^. ICC genes showed GO terms related to membrane excitability including membrane integrity, plasma membrane, metal ion binding, and transport^13^. PDGFRα^+^ cell genes revealed GO categories related to the function and structure of the extracellular matrix^12,14^.

The cell-specific markers identified in our previous transcriptome studies provide new tools to study SIP cells^11–13^ and our new SMTB allows for easy visualization and comparison of this elucidated data. We found that the most distinctive markers for detecting primary mature SMC are *Cnn1*, *Mylk*, *Tpm2*, *Tpm1*, *Des*, and *Myh11*^11^. SMC are phenotypically dynamic with the ability to dedifferentiate back to a myofibroblast-like state induced by pathological conditions causing SMC overgrowth and within *in vitro* conditions^24^. The six SMC markers should be used together to evaluate SMC phenotype in pathological conditions or cultured conditions. We also identified the new markers *Thbs4 and Hcn4* that could be used in ICC identification. Currently, and historically, ICC are identified and tagged through the use of KIT (CD117) antibodies. However, the expression of KIT is not consistent across various ICC phenotypic conditions^25^. For example, in some GI pathologies that cause digestive dysmotility, ICC lose their expression of KIT, making KIT antibodies a poor marker of ICC as they cannot be used to track ICC that undergo phenotypic alterations^26^. Our newly uncovered ICC-selective marker, THBS4, may allow for the tracking of ICC long after KIT expression is lost. Additionally, we also found that expression of *Cacna1g* may be an identifying marker of hyperplasic PDGFRα^+^ cells in the context of various GI diseases (small bowel obstruction, colorectal cancer, Crohn’s disease, and diverticulitis)^12^. Lastly, we identified the new maker *Adamdec1* in mucosal PDGFRα^+^ cells (unpublished data). Expression of *Adamdec1* is induced in the DSS-induced colitis mouse model and human tissue affected by Crohn’s disease (unpublished data).

Prior to the publication of our SIP cell transcriptomes, there was no transcriptome-level comparative tools available for analyzing cells contained within the SIP syncytium; it was only available to be compared through individual extraction of each data point in extremely large and generally opaque spreadsheets. Our new SMTB is a new and dynamic tool that allows for quick, easy, and clear-cut access to genetic information for individual transcripts in individual SIP cells and their associated tissue while allowing for comparison between both transcripts and cells/tissue. This tool will bring insights into new potential pathways for future research in the vast field of GI smooth muscle biology.

## Materials and Methods

### Intestinal tissues and cells

Pooled total RNAs were isolated from gastrointestinal (GI) tissue (2 males and 2 females, 1–2 months old) or sorted cells (up to 20 males and 20 females, 1–2 months old) from GFP reporter mouse lines: SMC from Myh11-Cre-eGFP^27^, ICC from Kit-copGFP^28^, and PDGFRα^+^ cells from Pdgfra-eGFP^29^ (Table 1). All procedures involving animals and their care were performed in accordance with institutional, state and national guidelines. The animal protocol was approved by the Institutional Animal Care and Use Committee at the University of Nevada-Reno Animal Resources. RNA-seq libraries were generated and sequenced via Illumina HiSeq 2000 (Illumina, San Diego, CA) at LC Sciences (Houston, TX) as previously described^11–14^.

### RNA-seq data analysis

Paired-end sequencing reads were isolated, processed, and analyzed as previously described^11–14^. An FPKM = 0.025 was selected as our cutoff expression value as it resulted in equal false positive and false negative ratios of reliability. Transcripts with FPKM values of less than 0.025 were considered to be 0. Sequencing reads were assembled and annotated onto a reference genome (UCSC mm9 or mm10) using TopHat v1.4.1 software.

### Transcriptome

Transcriptomes obtained from RNA-seq data analyses are shown in Table 2^11–14^. Briefly, we obtained total reads of 136.6 M–238.2 M, 77–93% of which were mapped to the murine genome. The mapping identified 46,299–55,345 transcriptional isoform variants, which were aligned to 15,192–17,172 known genes. The average number of transcriptional variants per gene was 3.

### Smooth Muscle Transcriptome Browser

Gbrowse 2.0^30^ was used to build the SMTB. Gbrowse is a web-based genome browser originally developed in 2002 for use with Wormbase^31^. It enables web-based graphical sequence displays and sequence annotation. The browser has been used for other public data sources such as Flybase^32^, SGD^33^, and SilkDB^34^. In the browser, data can be downloaded for all transcripts on transcript detail pages. The SMTB was created using the standard Gbrowse 2.0 install with custom modifications. In addition to configuration files, Gbrowse files modified included CSS files and the cgi scripts “gbrowse” and “gbrowse_details”. Gbrowse 2.0 was installed on a Linux Debian 7.6 operating system. Transcript data files were converted to gff3 format and then uploaded to a MySQL backend database included with the install. Images for each transcript containing expression levels and number of splice variants were created with in house software.

### Future Directions

We plan to expand the Smooth Muscle Transcriptome Sequencing Project to other GI cell types in mice and human GI cell types, as well as update the SMTB.

## Electronic supplementary material

Supplementary information

## Data Availability

The RNA-seq data from this study have been deposited to the NCBI Gene Expression Omnibus (GEO): GSM1388406, Jejunum SMC; GSM1388407, Colon SMC; GSM1388408, Jejunum ICC; GSM1388409, Colon ICC; GSM1388410, Jejunum PDGFRα^+^ cell; GSM1388411, Colon PDGFRα^+^ cell; GSM1388412, Jejunum SM; GSM1388413, Colon SM; GSM1388414, Colon Mu; GSM1388415, Colon Mu PDGFRα^+^ cell.

## References

[1] Sanders KM, Koh SD, Ro S, Ward SM (2012). Regulation of gastrointestinal motility–insights from smooth muscle biology. Nature reviews. Gastroenterology & hepatology.

[2] Sanders KM, Koh SD, Ward SM (2006). Interstitial cells of cajal as pacemakers in the gastrointestinal tract. Annual review of physiology.

[3] Bornstein JC, Costa M, Grider JR (2004). Enteric motor and interneuronal circuits controlling motility. Neurogastroenterology and motility: the official journal of the European Gastrointestinal Motility Society.

[4] Kurahashi M, Mutafova-Yambolieva V, Koh SD, Sanders KM (2014). Platelet-derived growth factor receptor-alpha-positive cells and not smooth muscle cells mediate purinergic hyperpolarization in murine colonic muscles. American journal of physiology. Cell physiology.

[5] Baker SA, Hennig GW, Ward SM, Sanders KM (2015). Temporal sequence of activation of cells involved in purinergic neurotransmission in the colon. The Journal of physiology.

[6] Martucciello G, Ceccherini I, Lerone M, Jasonni V (2000). Pathogenesis of Hirschsprung’s disease. J Pediatr Surg.

[7] Ordog T, Hayashi Y, Gibbons SJ (2009). Cellular pathogenesis of diabetic gastroenteropathy. Minerva gastroenterologica e dietologica.

[8] Miettinen, M. & Lasota, J. Gastrointestinal stromal tumors: review on morphology, molecular pathology, prognosis, and differential diagnosis. *Archives of pathology & laboratory medicine* **130**, 1466–1478, 10.1043/1543-2165(2006)130[1466:GSTROM]2.0.CO;2 (2006). 10.5858/2006-130-1466-GSTROM 17090188

[9] Speca S, Giusti I, Rieder F, Latella G (2012). Cellular and molecular mechanisms of intestinal fibrosis. World journal of gastroenterology: WJG.

[10] Stanghellini V, Corinaldesi R, Barbara L (1988). Pseudo-obstruction syndromes. Bailliere’s clinical gastroenterology.

[11] Lee MY (2015). Smooth Muscle Cell Genome Browser: Enabling the Identification of Novel Serum Response Factor Target Genes. PloS one.

[12] Ha SE (2017). Transcriptome analysis of PDGFRalpha+ cells identifies T-type Ca2+ channel CACNA1G as a new pathological marker for PDGFRalpha+ cell hyperplasia. PloS one.

[13] Lee MY (2017). Transcriptome of interstitial cells of Cajal reveals unique and selective gene signatures. PloS one.

[14] Ha, S. *et al*. Transcriptome profiling of subepithelial PDGFRα+ cells in colonic mucosa reveals several cell-selective markers (In review). 10.1371/journal.pone.0261743 PMC9106222 35560163

[15] Wheeler DL (2003). Database resources of the National Center for Biotechnology. Nucleic acids research.

[16] Mouse EC (2012). An encyclopedia of mouse DNA elements (Mouse ENCODE). Genome biology.

[17] Meyer LR (2013). The UCSC Genome Browser database: extensions and updates 2013. Nucleic acids research.

[18] Messeguer X (2002). PROMO: detection of known transcription regulatory elements using species-tailored searches. Bioinformatics.

[19] Pundir S, Martin MJ, O’Donovan C (2017). UniProt Protein Knowledgebase. Methods in molecular biology.

[20] Farkas MH (2013). Transcriptome analyses of the human retina identify unprecedented transcript diversity and 3.5 Mb of novel transcribed sequence via significant alternative splicing and novel genes. BMC genomics.

[21] Zhang Y (2014). An RNA-sequencing transcriptome and splicing database of glia, neurons, and vascular cells of the cerebral cortex. The Journal of neuroscience: the official journal of the Society for Neuroscience.

[22] Lee MY (2017). Serum response factor regulates smooth muscle contractility via myotonic dystrophy protein kinases and L-type calcium channels. PloS one.

[23] Kurahashi M (2008). Platelet-derived growth factor signals play critical roles in differentiation of longitudinal smooth muscle cells in mouse embryonic gut. Neurogastroenterology and motility: the official journal of the European Gastrointestinal Motility Society.

[24] Owens GK, Kumar MS, Wamhoff BR (2004). Molecular regulation of vascular smooth muscle cell differentiation in development and disease. Physiological reviews.

[25] Torihashi S (1999). Blockade of kit signaling induces transdifferentiation of interstitial cells of cajal to a smooth muscle phenotype. Gastroenterology.

[26] Vanderwinden JM, Rumessen JJ (1999). Interstitial cells of Cajal in human gut and gastrointestinal disease. Microscopy research and technique.

[27] Xin HB, Deng KY, Rishniw M, Ji G, Kotlikoff MI (2002). Smooth muscle expression of Cre recombinase and eGFP in transgenic mice. Physiological genomics.

[28] Ro S (2010). A model to study the phenotypic changes of interstitial cells of Cajal in gastrointestinal diseases. Gastroenterology.

[29] Hamilton TG, Klinghoffer RA, Corrin PD, Soriano P (2003). Evolutionary divergence of platelet-derived growth factor alpha receptor signaling mechanisms. Molecular and cellular biology.

[30] Stein LD (2013). Using GBrowse 2.0 to visualize and share next-generation sequence data. Brief Bioinform.

[31] Rogers A (2008). WormBase 2007. Nucleic acids research.

[32] Tweedie S (2009). FlyBase: enhancing Drosophila Gene Ontology annotations. Nucleic acids research.

[33] Hong EL (2008). Gene Ontology annotations at SGD: new data sources and annotation methods. Nucleic acids research.

[34] Duan J (2010). SilkDBv2.0: a platform for silkworm (Bombyx mori) genome biology. Nucleic acids research.

