# Supplementary material for: Smooth Muscle Transcriptome Browser: offering genome-wide references and expression profiles of transcripts expressed in intestinal SMC, ICC, and PDGFRα+ cells
Source: Sci Rep. 2019 Jan 23;9:387. doi: 10.1038/s41598-018-36607-6 (PMC6344548; doi:10.1038/s41598-018-36607-6)
Supplement: Supplementary file 1 — Supplementary information [file 41598_2018_36607_MOESM1_ESM.pdf]

Smooth Muscle Transcriptome Browser: offering genome-wide references and expression profiles of transcripts expressed in intestinal SMC, ICC, and PDGFRα$^+$ cells

Adrienne Breland[1,2], Se Eun Ha[1,2], Brian G. Jorgensen[1,2], Byungchang Jin[1], Treg A. Gardner[1], Kenton M. Sanders[1], Seungil Ro[1]

1. Department of Physiology and Cell Biology, University of Nevada School of Medicine, Reno, Nevada
2. These three authors contributed equally to this work

Supplementary Figures

a

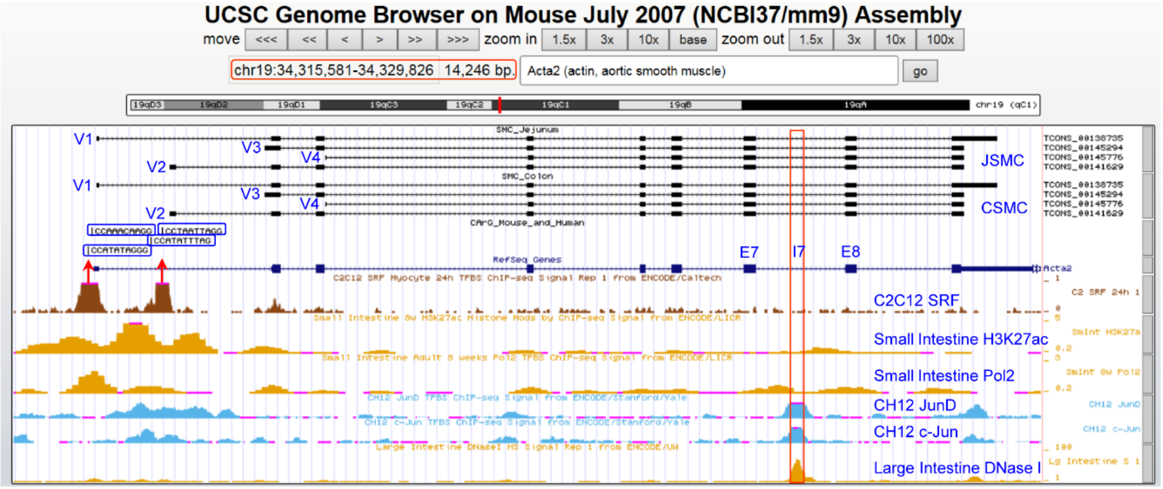

b

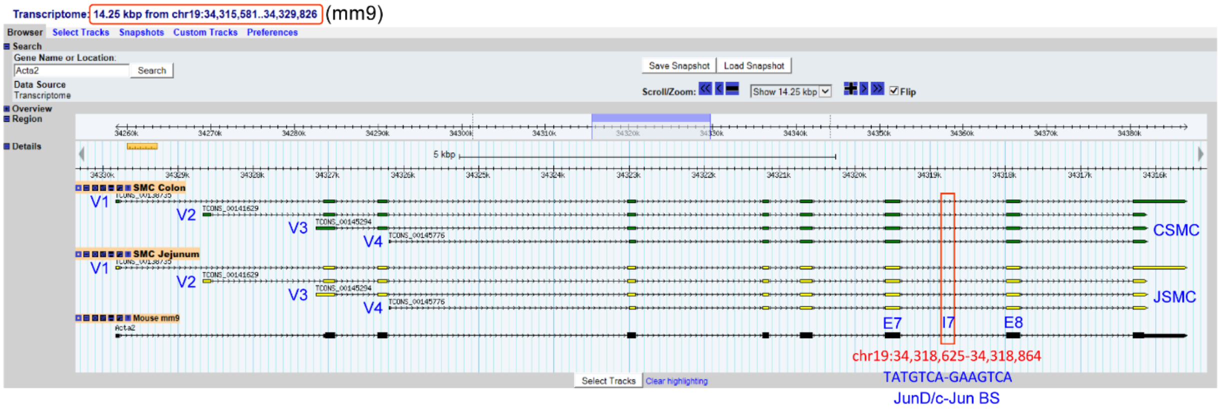

Figure S1. Complementary analysis of *Acta2* using both the Smooth Muscle Genome Browser (SMGB) and Smooth Muscle Transcriptome Browser (SMTB). (**a**) A map view of *Acta2* transcripts in the SMGB. Four transcriptional variants (V1-4) identified in CSMC and JSMC are shown within chr19:34,315,581-34,329,826 in the mouse mm9 genome. Four CArG boxes that are conserved between mice and humans are boxed in blue. Two SRF binding sites match with the CArG boxes proximate to V1 and V2 first exons. The promoter region coincides with H3K27ac modification sites and Pol2 binding sites in the small intestine genome. JunD and c-Jun binding sites are shown within intron 7. These Jun binding sites coincide with the DNase I hypersensitive region in the large intestine genome. (**b**) A map view of *Acta2* transcripts in the SMTB. The same four transcriptional variants (V1-4) identified in CSMC and JSMC are shown contained within a 14.25 kb segment (chr19:34,315,581-34,329,826 in mm9). The two JunD and c-Jun binding sites, TATGTCA and GAAGTCA, are found at chr19:34,318,625-34,318,864. For all ENCODE data displays (Maximum display mode, full; Peaks, hide; Signal, full), the following data sets were selected in Expression and Regulation: for C2C12 SRF, Caltech TFBS; for Small Intestine H3K27ac, LICR Histone; for Small Intestine Pol2, LICR TFBS; for CH12 JunD and CH12 c-Jun, Stan/Yale TFBS; for Large Intestine DNase I, UW DNase I HS.

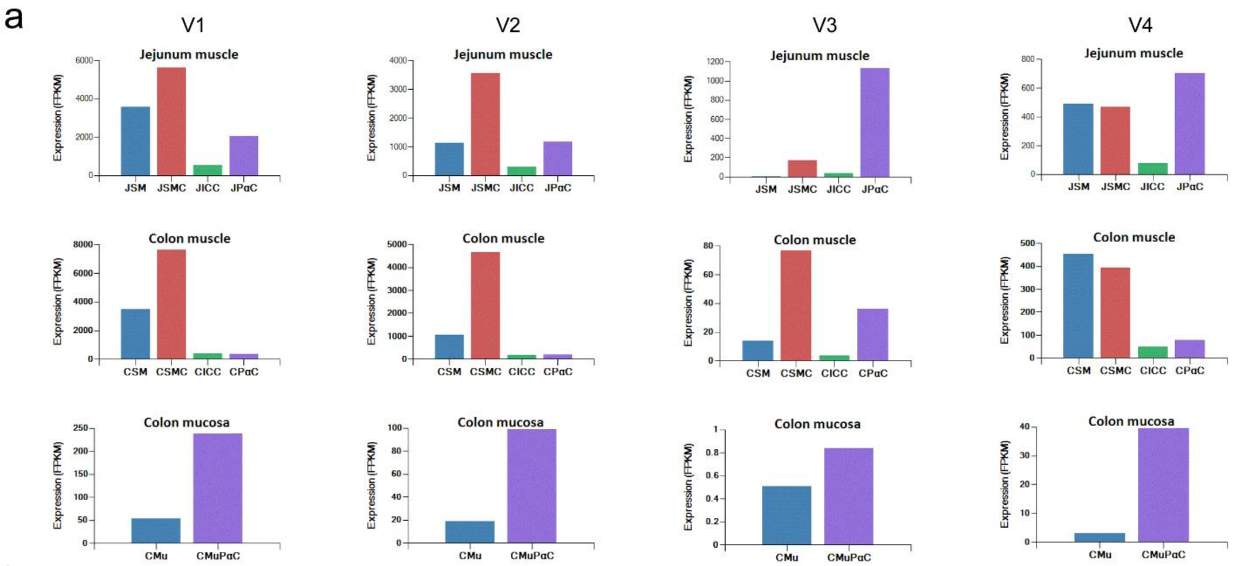

b

```
   23                                      46 49                         75         86
V1 MCEEEDSTALVCDNGSGLCKAGFAGDDAPRAVFPSIVGRPRHQGVMVGMGQKDSYVGDEAQSKRGILTLKYPIEHGIITNWDDMEKIWHHSFYNELRVAPEEHPTLLTEAPLNPKANREK 120
V2 MCEEEDSTALVCDNGSGLCKAGFAGDDAPRAVFPSIVGRPRHQGVMVGMGQKDSYVGDEAQSKRGILTLKYPIEHGIITNWDDMEKIWHHSFYNELRVAPEEHPTLLTEAPLNPKANREK 120
V3 MCEEEDSTALVCDNGSGLCKAGFAGDDAPRAVFPSIVGRPRHQGVMVGMGQKDSYVGDEAQSKRGILTLKYPIEHGIITNWDDMEKIWHHSFYNELRVAPEEHPTLLTEAPLNPKANREK 120
V4 ------------------------------------------------------------------------------------------------------------------------ 0

V1 MTQIMFETFNVPAMYVAIQAVLSLYASGRTTGIVLDSGDGVTHNVPIYEGYALPHAIMRLDLAGRDLTDYLMKILTERGYSFVTTAEREIVRDIKEKLCYVALDFENEMATAASSSSLEK 240
V2 MTQIMFETFNVPAMYVAIQAVLSLYASGRTTGIVLDSGDGVTHNVPIYEGYALPHAIMRLDLAGRDLTDYLMKILTERGYSFVTTAEREIVRDIKEKLCYVALDFENEMATAASSSSLEK 240
V3 MTQIMFETFNVPAMYVAIQAVLSLYASGRTTGIVLDSGDGVTHNVPIYEGYALPHAIMRLDLAGRDLTDYLMKILTERGYSFVTTAEREIVRDIKEKLCYVALDFENEMATAASSSSLEK 240
V4 MTQIMFETFNVPAMYVAIQAVLSLYASGRTTGIVLDSGDGVTHNVPIYEGYALPHAIMRLDLAGRDLTDYLMKILTERGYSFVTTAEREIVRDIKEKLCYVALDFENEMATAASSSSLEK 120
   ************************************************************************************************************************

V1 SYELPDGQVITIGNERFRCPETLFQPSFIGMESAGIHETTYNSIMKCDIDIRKDLYANNVLSGGTTMYPGIADRMQKEITALAPSTMKIKIIAPPERKYSVWIGGSILASLSTFQQMWIS 360
V2 SYELPDGQVITIGNERFRCPETLFQPSFIGMESAGIHETTYNSIMKCDIDIRKDLYANNVLSGGTTMYPGIADRMQKEITALAPSTMKIKIIAPPERKYSVWIGGSILASLSTFQQMWIS 360
V3 SYELPDGQVITIGNERFRCPETLFQPSFIGMESAGIHETTYNSIMKCDIDIRKDLYANNVLSGGTTMYPGIADRMQKEITALAPSTMKIKIIAPPERKYSVWIGGSILASLSTFQQMWIS 360
V4 SYELPDGQVITIGNERFRCPETLFQPSFIGMESAGIHETTYNSIMKCDIDIRKDLYANNVLSGGTTMYPGIADRMQKEITALAPSTMKIKIIAPPERKYSVWIGGSILASLSTFQQMWIS 240
   ************************************************************************************************************************

V1 KQEYDEAGPSIVHRKCF 377
V2 KQEYDEAGPSIVHRKCF 377
V3 KQEYDEAGPSIVHRKCF 377
V4 KQEYDEAGPSIVHRKCF 257
   *****************
```

Figure S2. Comparison of mRNA expression and *in silico* translated amino acid sequence of *Acta2* variants in intestinal tissue and cells. (**a**) mRNA expression of *Acta2* variants V1-V4 in Jejunal smooth muscle tissue (JSM), isolated jejunal cells (JSMC, JICC, and JPαC), colonic smooth muscle tissue (CSM) and isolated colonic cells (CSMC, CICC, and CPαC), as well as colonic mucosa tissue (CMu) and isolated cells (CMuPαC). (**b**) Alignment of *in silico* translated primary amino acid sequence of variant open reading frames. Sequence beginning methionines (M) found at the origin of V1-4 are indicated in red. Post-translationally modified residues (2, 3, 46, 49, 75, and 86 from UniProt are highlighted in green.
